# Assessing the Contribution of Heme-Iron Acquisition to S*taphylococcus aureus* Pneumonia Using Computed Tomography

**DOI:** 10.1371/journal.pone.0006668

**Published:** 2009-08-18

**Authors:** William Jeffrey Mason, Eric Patrick Skaar

**Affiliations:** 1 Division of Infectious Diseases, Department of Internal Medicine, Vanderbilt University School of Medicine, Nashville, Tennessee, United States of America; 2 Department of Microbiology and Immunology, Vanderbilt University School of Medicine, Nashville, Tennessee, United States of America; BMSI-A*STAR, Singapore

## Abstract

**Background:**

*S. aureus* acquires heme-iron using the iron regulated surface determinant (Isd) system and the heme transport system (Hts) with both systems showing critical importance in systemic models of infection. The contribution of heme-iron acquisition to staphylococcal pneumonia has not yet been elucidated. In addition, the use of computed tomography (CT) for the evaluation of staphylococcal pneumonia and its correlation to pathologic examination of infected lung tissue has not been performed to date. We have applied CT-based imaging to a murine model of staphylococcal pneumonia to determine the virulence contribution of heme-iron acquisition through the Hts and Isd systems.

**Methodology/Principal Findings:**

Mice were intranasally inoculated with ∼1.0×10^8^ colony forming units (CFU) of *S. aureus*. Lungs from mice infected with wild type *S. aureus* or strains deficient in *isdB* and *isdH* (*ΔisdBH*) or *htsA* and *isdE* (Δ*htsAΔisdE*) were harvested at 24 hours. Histology, radiographic appearance by computed tomography (CT), percent mortality and bacterial burden were evaluated. Infection with *S. aureus ΔisdBH* and *ΔhtsAΔisdE* did not result in a statistically significant difference in mortality or bacterial burden as compared to controls. CT imaging of infected mice also did not reveal an appreciable difference between the various strains when compared to wild type, but did correlate with pathologic findings of pneumonia. However, a systemic model of infection using the *ΔhtsAΔisdE* strain revealed a statistically significant decrease in bacterial burden in the lung, heart and kidneys.

**Conclusions/Significance:**

The development of staphylococcal pneumonia in this murine model is not dependent on hemoglobin binding or heme-iron uptake into *S. aureus*. However, this model does reveal that heme-iron acquisition contributes to the pathogenesis of systemic staphylococcal infections. In addition, CT imaging of murine lungs is an attractive adjunct to histologic analysis for the confirmation and staging of pneumonia.

## Introduction

Bacteria require iron as a nutrient source and almost all bacterial pathogens are incapable of causing disease without iron. *S. aureus* satisfies its nutrient iron requirement through the acquisition of heme-iron by the iron regulated surface determinant (Isd) system and the heme transport system (Hts) [1], [2]. The Hts and Isd systems employ a series of cell wall anchored proteins (IsdA, IsdB, IsdC and IsdH), two membrane transporters (IsdDEF, HtsABC), and two cytoplasmic heme oxygenases (IsdG and IsdI) to acquire heme-iron from vertebrate hemoglobin. The model for Isd and Hts-mediated heme-iron acquisition initiates upon hemoprotein binding to the cell wall receptors IsdB and IsdH. Following hemoprotein capture, heme is removed through as-yet-unidentified mechanisms and passed to IsdA and IsdC. IsdA and IsdC then deliver heme to IsdDEF and HtsABC for passage into the cytoplasm where heme is degraded by IsdG and IsdI to release free iron. IsdDEF is a membrane transporter composed of solute-binding proteins (IsdD and IsdE) and a permease (IsdF) whereas HtsABC is an ABC-type transporter made up of a solute-binding protein (HtsA) and a dual protein permease (HtsB and HtsC). Inactivation of individual components of IsdDEF and HtsABC affects heme import into *S. aureus* suggesting that the systems are redundant at some level for heme acquisition. In addition, both the Hts and Isd systems are individually required for full virulence in systemic models of staphylococcal infection [2]–[4] however their combined contribution to pathogenesis has not yet been elucidated.

Despite the known importance of heme-iron during systemic staphylococcal infection, the contribution of heme-iron acquisition to the pathogenesis of staphylococcal pneumonia has not been evaluated. It has been previously shown that *S. aureus* removed from infected murine lungs are coated with considerable levels of host hemoglobin suggesting that heme-iron may be a relevant iron source at this anatomical site [5]. A potential reason for this gap in knowledge regarding the nutrient iron sources utilized during staphylococcal pneumonia is technical limitations associated with studying pneumonia in a murine model. The development of improved techniques to monitor the progression of pneumonia in murine models would increase our understanding of the pathophysiology of this disease.

Plain film chest radiography is the most commonly used radiographic technique for the diagnosis of pneumonia. However, computed tomography (CT) may be employed in situations where the diagnosis remains in question or if complications are suspected. CT imaging is far superior to plain film chest radiography at determining the specific anatomic location and distribution pattern of pneumonia [6]. CT has been used to correlate pathologic findings in a murine model of *Pasteurella* pneumonia [7]. However, CT imaging has yet to be used to investigate the contribution of virulence factors to the radiographic appearance of murine lungs during bacterial pneumonia. Further, CT imaging has not been used to study the development of staphylococcal pneumonia in murine models. Therefore, we sought to apply CT-based imaging techniques to study the contribution of heme-iron acquisition to staphylococcal pneumonia.

## Materials and Methods

*S. aureus* Newman was used for all experiments and mutants in the Hts and Isd system were created in this strain background. The Δ*isdBH* mutant used in this study has been previously described [8]. The *ΔhtsAΔisdE* mutant was created for this study using allelic replacement procedures described by Bae and Schneewind [9]. This mutagenesis procedure resulted in the creation of *S. aureus* single mutant strains containing a deletion of *htsA* (Δ*htsA*) or a replacement of *isdE* with an erythromycin resistance marker (Δ*isdE*). To create a strain simultaneously inactivated for both *htsA* and *isdE*, the *isdE* mutant allele was mobilized into Δ*htsA* using phage transduction with Φ85. This *ΔhtsAΔisdE* mutant is functionally inactivated for both Isd and Hts-mediated heme transport. No growth differences were observed between *S. aureus* wild type and the various mutant strains used in this study when grown in rich medium suggesting that these strains do not exhibit general growth defects in iron replete medium (data not shown). Inoculation doses for the mouse model of pneumonia were created by diluting an overnight culture of each strain 1∶100 followed by horizontal incubation at 37 degrees Celsius (°C) with 180 rotations per minute (rpm) of shaking. Following 3 hours of growth, the bacteria were pelleted at 5,900 g for 6 minutes and then washed two times with endotoxin free phosphate buffered saline (PBS). The resultant pellet was then resuspended in a fixed volume of endotoxin free PBS and analyzed spectrophotometrically at 600 nm. Triplicate dilutions of each inoculation dose were performed in sterile tryptic soy broth (TSB) and enumerated on tryptic soy agar (TSA). Inoculation doses for the murine systemic model of infection were created by diluting an overnight culture of each strain 1∶100 in 5 mls of sterile TSB followed by horizontal incubation at 37 °C with 180 rpm of shaking. After 3 hours of growth, the bacteria were pelleted at 5,900 g for 6 minutes and resuspended in endotoxin free PBS to a final optical density of 0.4 (600 nm).

Following anesthesia, 7 week old BALB/c mice (Jackson Laboratories) were infected with 30 µl of the bacterial slurry into the right nare and held upright for sixty seconds. Mice were euthanized if they experienced increased lethargy, decreased mobility, a decrease in resistance to handling, or any other concerning signs or symptoms suggesting that they might succumb before the 24 hour period. At 24 hours post-infection, mice were euthanized using forced inhaled CO_2_. The lungs were aseptically explanted, homogenized in 1 ml of sterile PBS, and colony forming units were enumerated on TSA. For histology, the trachea was incised and intubated with a blunt end needle while the mainstem bronchus of each lung was isolated. The lungs were inflated with 1.0 ml of 10% formalin and each mainstem bronchus was tied off. They were then submerged in 10% formalin, stored in 70% ethanol and stained with hematoxylin and eosin (H and E). For the systemic model of infection, 7 week old BALB/c mice were retroorbitally infected with approximately 1.5×10^7^ colony forming units (CFU) of each staphylococcal strain. Ninety-six hours post infection, the mice were euthanized and the lungs, hearts, kidneys, spleens and livers were explanted and homogenized using 1 ml of sterile PBS. The bacteria were enumerated on TSA. The animal protocols used in this study were approved by the Vanderbilt University Medical Center (VUMC) Institutional Animal Care and Use Committee (IACUC). Statistical analysis was performed using a Student's *t* test in bacterial burden experiments.

For murine CT, mice were imaged in the microCAT II (Siemens, Knoxville, TN) at an x-ray tube voltage of 80 kVp and a tube current of 0.5 mA. The exposure time was set to 300 ms per projection and the gantry was rotated over 360 degrees while 601 two-dimensional projections were taken. The images were reconstructed using a Hamming filter into transaxial slices of 512×512×512 with voxel sizes of 0.124×0.124×0.062 mm^3^.

## Results

**Figure 1:**
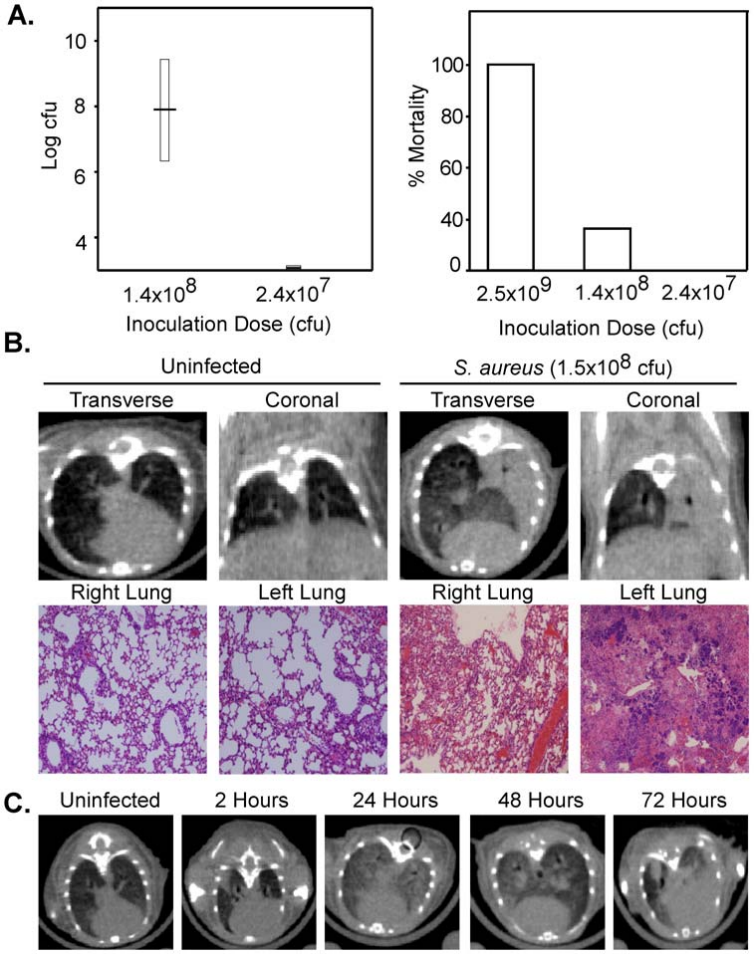
The dose dependency and radiographic appearance of *S. aureus* pneumonia. (A): Bacterial burden and percent mortality during *S. aureus* pneumonia following 24 hours post intranasal (i.n.) infection. The horizontal bar in the left most figure represents the average bacterial burden presented as colony forming units of homogenized lung tissue with the boxes representing the standard deviation of the sample population. (B): Murine CT imaging correlated with pulmonary pathology at 24 hours. (C): Murine serial CT imaging of a single mouse following i.n. inoculation with 3.7×10^8^ CFU *S. aureus*.

In order to develop a murine model of staphylococcal pneumonia, mice were intranasally infected with either 2.4×10^7^, 1.4×10^8^, or 2.5×10^9^ CFU and monitored for 24 hours. The bacterial burden of homogenized lung tissues and percent mortality were calculated at the conclusion of the 24 hour period (Figure 1A). Pathologic lesions consistent with pneumonia were not seen with the 2.4×10^7^ CFU inoculation dose. However, pathologic lesions consistent with pneumonia were observed with the 1.4×10^8^ and 2.5×10^9^ CFU doses (data not shown). Based on the high level of mortality seen with the 2.5×10^9^ CFU dose, we used an inoculation dose of approximately 1.0×10^8^ CFU for subsequent experiments. This inoculum is consistent with those used in other published intranasal staphylococcal pneumonia models [10].

CT imaging was performed on uninfected and infected animals and correlated to H and E sections of the respective lungs at 24 hours. Uninfected animals showed no radiographic or pathologic evidence of pneumonia. In contrast, animals infected with 1.5×10^8^ CFU showed a dense consolidation of the left lung consistent with pneumonia and pathologic analysis of the left lung confirmed this radiographic finding (Figure 1B). In addition, the radiographic progression of *S. aureus* pneumonia over a 72 hour period revealed a progressive focal infiltrate in the bilateral lung fields (Figure 1C). These data show that CT imaging of murine models of staphylococcal pneumonia is an attractive adjunct technique to pathologic analysis.

**Figure 2:**
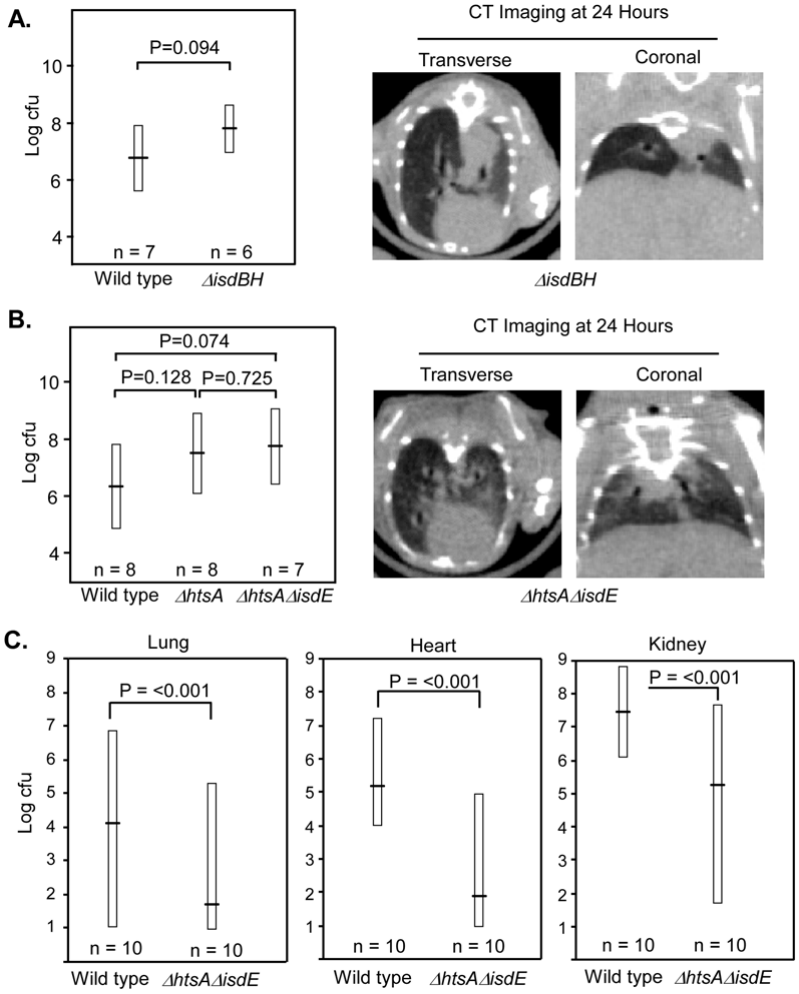
The contribution of heme acquisition to the virulence of *S. aureus*. (A): Bacterial burden and CT imaging of *S. aureus* wild type vs. *S. aureus ΔisdBH* pneumonia at 24 hours post infection. The horizontal bar represents the average bacterial burden in colony forming units of homogenized lung tissue with the boxes representing the standard deviation of the sample population. Sample sizes are indicated below each bar. (B): Bacterial burden and CT imaging of *S. aureus* pneumonia at 24 hours post infection with wild type, *ΔhtsA* or *ΔhtsAΔisdE*. The horizontal bar in the left most figure represents the average bacterial burden in colony forming units of homogenized lung tissue with the boxes representing the standard deviation of the sample population. Sample sizes are indicated below each sample. (C): Bacterial burden in homogenized lung, heart and kidney in a systemic model of staphylococcal infection comparing *S. aureus* wild type to *S. aureus ΔhtsAΔisdE*. Sample sizes are indicated below each bar.

The contribution of heme-iron acquisition to staphylococcal pneumonia was evaluated by infecting mice with staphylococcal strains unable to bind hemoglobin (*ΔisdBH*) and strains unable to import heme into the staphylococcal cytoplasm (*ΔhtsAΔisdE*). The average bacterial burden of homogenized lung tissues and the radiographic appearance by CT imaging was not significantly different between the strains (Figure 2A and 2B). The mortality for these experiments was similar to the observed mortality seen with an inoculation dose of 1.4×10^8^ CFU wild type (Figure 1A) for each strain utilized.

Heme-iron acquisition is critically important to staphylococcal pathogenesis in systemic models of infection. Specifically, strains inactivated for *isdBH* [8], or *htsB* and *htsC* [2] exhibit pronounced virulence defects in a kidney abscess model of infection. However, the combined contribution of the Isd and Hts systems to systemic staphylococcal infections has not been evaluated. To determine the virulence contribution of heme transport through the staphylococcal membrane, BALB/c mice were infected retroorbitally with *S. aureus* wild type and *ΔhtsAΔisdE*. These experiments revealed a statistically significant decrease in the bacterial burden in the lung, heart and kidney of *ΔhtsAΔisdE* as compared to wild type (Figure 2C). Taken together, these results reveal that heme transport through the Isd and Hts systems is critical to the pathogenesis of systemic infection but dispensable in a murine model of pneumonia.

## Discussion

In this study, we have shown that CT imaging is an attractive adjunct to histological analysis for studying staphylococcal pneumonia in a murine model and could potentially be used as an alternative to histological analysis under certain situations. The fact that euthanasia, tissue explanation, and preservation are requirements for histological analysis confounds the ability to accurately capture the true pathologic process. In contrast, CT employs a noninvasive means of accurately assessing the development of pneumonia without requiring euthanasia [7]. In addition, CT may be used to follow the progression of pneumonia in a single animal and therefore allow researchers to track disease development over time as opposed to only viewing a single time point following euthanasia. These qualities of CT-based imaging provide increased resolution to animal models of pneumonia while allowing researchers to decrease the number of animals used.

In the murine staphylococcal pneumonia model used for this study, the heme-iron acquisition systems Isd and Hts had little influence on the development or pathogenesis of pneumonia. This may be due to the fact that staphylococci are able to acquire iron from other host sources when colonizing this anatomical site. Alternatively, the short duration of this infection model may not necessitate that *S. aureus* acquire significant levels of iron in order to cause disease. It is also possible that the myriad of staphylococcal virulence factors involved in the pathogenesis of this disease may mask the importance of iron acquisition during pneumonia. Despite the lack of involvement of Isd/Hts during the pathogenesis of pneumonia, these studies establish that *S. aureus* strains unable to acquire iron through either the Hts or Isd systems are severely decreased in virulence in a systemic model of infection. This finding implies that *S. aureus* require the acquisition of heme-iron at some point during dissemination in order to establish and maintain infection. Taken together, these results suggest that colonization of distinct anatomic sites involves the utilization of different iron sources. Additional work is needed to determine the specific iron sources utilized by *S. aureus* during lung colonization, and the contribution of virulence factors to nutrient iron acquisition in this anatomical site. Considering the significant virulence defect of staphylococcal strains lacking the Isd and Hts systems, heme-iron acquisition systems represent an attractive target for the generation of novel therapeutics to treat systemic staphylococcal infection.
